# Cu-MOFs Nanozymes with Ascorbate Oxidase and Peroxidase-like Activity for Sensitive Fluorometric Detection of Total Antioxidant Capacity in Fruits

**DOI:** 10.3390/nano16110665

**Published:** 2026-05-25

**Authors:** Yanyan Huang, Jing Chen, Ai Nasi, Yiming Zhao, Xin Ding, Dan Xu, Fengzhi Lyu, Donghui Xu, Meng Zhang, Ge Chen, Guangyang Liu

**Affiliations:** 1 National Center of Technology Innovation for Comprehensive Utilization of Saline-Alkali Land, 8 Zhihui Road, Agricultural High Tech Industry Demonstration Zone, Yellow River Delta, Dongying 257347, China; 2 State Key Laboratory of Vegetable Biobreeding, Institute of Vegetables and Flowers, Chinese Academy of Agricultural Sciences, Key Laboratory of Vegetables Quality and Safety Control, Ministry of Agriculture and Rural Affairs of China, Beijing 100081, China; 3 Key Laboratory of Geriatric Nutrition and Health, Ministry of Education, Beijing Technology & Business University, Beijing 100048, China

**Keywords:** Cu-MOFs, nanozymes, fruits, total antioxidant capacity

## Abstract

In this work, two-dimensional copper-based metal–organic frameworks (Cu-MOFs) nanozymes, including cuprous oxide-tetrakis (4-carboxyphenyl) porphyrin (Cu_2_O-TCPP) and copper-cuprous oxide-tetrakis (4-carboxyphenyl) porphyrin (Cu-Cu_2_O-TCPP), were synthesized, which exhibit dual ascorbate oxidase (AO) and peroxidase (POD)-like activities. The reductants, such as ascorbic acid (AA), can be oxidized by the cascade AO and POD catalysis on Cu-MOFs to oxidize p-phthalic acid (PTA) and generate fluorescence. Consequently, a fluorescence sensing platform for AA and other reducing substances was established. This platform offers potential for efficient and selective monitoring of reductive species and related antioxidant levels in food systems. The results showed that the two Cu-MOFs displayed favorable linear relationships (R^2^ ≥ 0.99) for the detection of AA, glutathione (GSH) and L-cysteine (L-Cys). Their limits of detection (LOD) were 5.3 μM for Cu_2_O-TCPP and 92.5 μM for Cu-Cu_2_O-TCPP. Finally, by detecting real samples of vitamin C tablets and fruits, the accuracy of the two Cu-MOFs nanos enzymes was validated, with Cu_2_O-TCPP showing higher accuracy.

## 1. Introduction

Antioxidants are important bioactive substances that help maintain human health and delay oxidative damage, with extensive applications in clinical practice [1,2], health management [3,4], and food science [5,6]. Natural antioxidants are widely found in various foods such as vegetables, fruits, herbs, and tea leaves [7,8,9]. By neutralizing free radicals, antioxidants contribute to anti-aging, disease prevention, and health maintenance, while also playing a significant role in food preservation [10,11,12]. Therefore, evaluating the antioxidant capacity of different foods is crucial for establishing scientific and rational dietary guidelines for health.

Total antioxidant capacity (TAC) is a key parameter for evaluating the antioxidant performance of foods. Currently, methods for measuring TAC include ORAC [13], FRAP [14], ABTS [14,15], DPPH [15], TEAC [13,16], CUPRAC [17], spectrophotometric assays [18], ferricyanide/Prussian blue assay [19], and others. However, these techniques often face limitations such as poor portability, lengthy detection times, and restrictions related to natural enzymes [20,21,22]. In recent years, platforms based on nanoenzymes have been developed for the assessment of TAC, providing more accurate and efficient evaluation of the antioxidant properties in food samples.

In recent years, with the rapid development of nanotechnology, nanozymes based on nanomaterials have demonstrated promising application prospects. Nanozymes have increasingly emerged as effective alternatives to natural enzymes due to their low cost and excellent thermal stability [23,24,25]. Among them, cascade-catalyzed nanozymes exhibit a series of clear advantages in catalytic synergy, substrate utilization, and signal transduction promotion [26,27]. Currently, materials used for the synthesis of nanozymes include graphene oxide [28,29,30], noble metal nanoparticles [31,32], carbon-based nanomaterials [33,34,35], and metal–organic frameworks (MOFs) [36,37,38].

MOFs are crystalline materials constructed from metal ions or clusters coordinated with organic ligands [39], forming inorganic–organic hybrid structures [40]. These materials exhibit high design flexibility, tunable pore architectures, and diverse structural features, enabling the mimicry of natural enzyme active sites and hydrophobic domains [38,41]. These characteristics endow MOF-based nanozymes with a large specific surface area, excellent pore structures, and good biocompatibility, thereby significantly enhancing their catalytic performance [40,42,43]. Currently, MOF-based nanozymes exhibit a variety of redox enzyme activities, including Oxidase-like nanozymes [44,45,46], Laccase-like nanozymes [47,48,49], Peroxidase-like nanozymes [40,50,51,52], Ascorbate oxidase nanozyme [53] and Superoxide dismutase-like nanozyme [54,55,56], and are increasingly applied in the fields of biomedicine [57,58,59], environmental science [60,61], and the food industry [62,63,64]. Among various functional ligands, tetrakis(4-carboxyphenyl)porphyrin (TCPP) is a typical porphyrin ligand with a rigid planar conjugated structure and abundant carboxyl groups, which can easily coordinate with metal ions to construct stable MOF materials. It possesses excellent electron transfer ability and biomimetic enzyme active center structure, thus endowing TCPP-based MOFs with outstanding catalytic activity, good structural stability, and favorable biocompatibility, and is widely used in the design and preparation of high-performance nanozymes.

This study synthesized two types of MOF-based nanozymes, which possess the cascade catalytic activity of POD and AO. AA and other reductive substances are initially oxidized by the AAO enzyme, producing hydroxyl radicals (·OH). Subsequently, PTA is oxidized by the ·OH radicals under POD catalysis, forming 2-hydroxy-1,4-benzenedicarboxylic acid, which fluoresces with excitation at 315 nm and emission at 430 nm. By utilizing this principle, the content of reductive (antioxidant) substances in the sample can be detected, with the TAC expressed in millimolar equivalents of AA per liter. This detection platform provides a new method for accurate and rapid detection of TAC in food.

## 2. Experimental Section

### 2.1. Materials and Instruments

Fluorescence intensity was measured using an RF-5301PC fluorescence spectrophotometer (Shimazu Corporation, Kyoto, Japan). Details of other reagents and instruments are listed in Table S1.

### 2.2. Preparation of Nanozyme

Preparations of Cu_2_O: CuSO_4_·5H_2_O (375 mg) and sodium citrate (147 mg) were dissolved in 80 mL of ultrapure water and were stirred magnetically for 15 min. NaOH (20 mL, 1.25 M) was added, and AA (50 mL, 0.03 M) was added after stirring for 15 min, stirred for 3 min, and then allowed to stand, centrifuged, and washed three times alternately with deionized water and ethanol, freeze-dried, and ground to obtain Cu_2_O powder.

The preparation of Cu-Cu_2_O: CuCl_2_ was stirred for 20 min after adding NaOH (5 mL, 0.2 M) under magnetic stirring (5 mL, 0.1 M). Ethanol (0.15 mL) was added, and then NaBH_4_ (25 mL, 2 mg/mL) aqueous solution was quickly added and stirred for 20 min, and the precipitate was washed twice with deionized water and ethanol alternately, freeze-dried, and ground to obtain Cu-Cu_2_O powder. Finally, Cu_2_O-TCPP and Cu-Cu_2_O-TCPP were synthesized by in situ polymerization.

### 2.3. Characterization

Scanning electron microscopy (SEM) and projection electron microscopy (TEM) were used to characterize the morphology of Cu_2_O-TCPP and Cu-Cu_2_O-TCPP. X-ray diffraction (XRD) was used to analyze the crystal structures of Cu_2_O-TCPP and Cu-Cu_2_O-TCPP. The X-ray photoelectron spectra (XPS), Brunauer–Emmett–Teller (BET) surface area, and N_2_ adsorption–desorption isotherms, thermogravimetric characterization (TGA), and Fourier transform infrared spectra (FTIR) of Cu_2_O-TCPP and Cu-Cu_2_O-TCPP were also analyzed.

### 2.4. AAO Activity of Cu_2_O-TCPP and Cu-Cu_2_O-TCPP and Condition Optimization

PTA was used as an enzyme catalytic substrate to evaluate the AAO-like activity of Cu_2_O-TCPP and Cu-Cu_2_O-TCPP. In order to determine the optimal reaction conditions for the detection of AA by Cu_2_O-TCPP and Cu-Cu_2_O-TCPP, the fitting of the fluorescence intensity of AA with AA concentration in the range of 0–0.5 mM was selected as the standard, and the concentrations of Cu_2_O-TCPP and Cu-Cu_2_O-TCPP, the pH value of the buffer solution and the concentration of PTA in the reaction system were optimized, and the reaction conditions with high R^2^ value and slope were finally determined as the optimal conditions. After determining the optimal detection conditions, the LOD of the sensing system was calculated using the classic 3σ method. The standard deviation was obtained by repeatedly measuring the fluorescence signal of blank samples, and the LOD was finally calculated by combining with the slope of the standard curve.

In order to determine the affinity of Cu_2_O-TCPP and Cu-Cu_2_O-TCPP for AA, the AA concentration range was selected to be 0–2 mM under the optimal reaction conditions, and the fluorescence intensity was detected at Ex = 315 nm immediately after the reaction was completed, 45 min, and 6 min after the solution was mixed. Considering the particularity of cascade nanozyme systems, the obtained kinetic parameters are only apparent values rather than standard intrinsic constants. In this work, we only used the Michaelis–Menten equation to fit the relationship between reaction rate and substrate concentration, and the acquired *Vmax* and *Km* are merely apparent kinetic parameters for horizontal comparison.

$$\frac{1}{v}=\frac{K_{m}}{V_{max}}\times \frac{1}{\left[S\right]}+\frac{1}{V_{max}}$$

where *v* is the initial reaction velocity, *Vmax* is the maximum reaction velocity, *[S]* is the substrate concentration, and *Km* is the Michaelis constant.

### 2.5. Antioxidant Fluorescence Detection

Cu_2_O-TCPP was reacted with PBS buffer (0.1 M, pH = 7.5, 6 mL), PTA (45 mM, 0.5 mL), Cu_2_O-TCPP (0.45 mg/mL) and AA (0.5 mM, 0.5 mL), and Cu-Cu_2_O-TCPP was reacted with PBS buffer (0.1 M, pH = 6.5, 6 mL), PTA (45 mM, 0.5 mL), Cu_2_O-TCPP (0.45 mg/mL) and AA (0.5 mM, 0.5 mL), and the reaction system was mixed and reacted at 45 °C for 15 min, and the fluorescence intensity was detected at Ex = 315 nm. Controls were set up to verify the enzymatic activities of Cu_2_O-TCPP and Cu-Cu_2_O-TCPP. In addition, the linear relationship between AA, GSH and L-Cys and fluorescence intensity was analyzed under optimal reaction conditions.

### 2.6. Actual Samples

In order to test the practical feasibility of the two nanozymes, the TAC values of the actual samples were measured. First, drugstore-bought vitamin C tablets were ground into a powder, dissolved in purified water, and prepared into a solution of 0.176 g/L. Secondly, the kiwifruit, orange and grapefruit were removed from the epidermis and beaten into a homogenate, the supernatant was obtained by centrifugation, and the vitamin C tablet solution and supernatant were filtered with a 0.2 mm microporous filter membrane for later use, and the concentration of the sample was diluted to within the linear range of AA detection.

PBS (0.1 M, 6 mL), PTA (45 mM, 0.5 mL), Cu_2_O-TCPP/Cu-Cu_2_O-TCPP (0.5 mL), and AA (0.5 mL) reactions were used to detect TAC with the actual sample filtrate instead of AA. The fluorescence intensity was detected under the optimal reaction conditions, with Ex = 315 nm and Em = 430 nm. Bring the fluorescence intensity into the corresponding standard curve and multiply it by the dilution factor to calculate the TAC content of the sample. The TAC of the actual sample is expressed in mM equivalents per liter of AA.

## 3. Results and Discussion

### 3.1. Characterization of Cu_2_O-TCPP and Cu-Cu_2_O-TCPP

**Figure 1:**
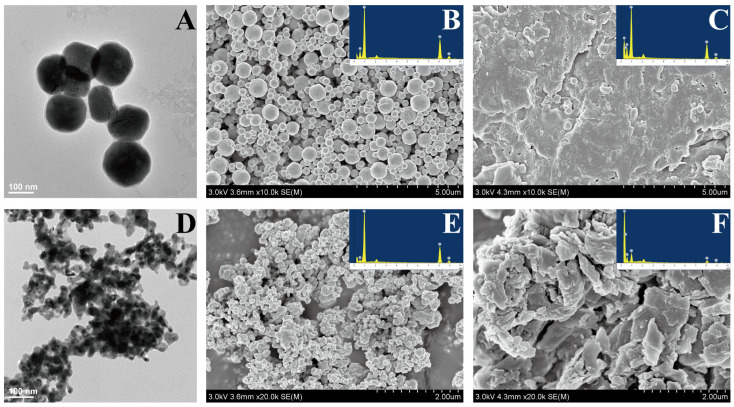
TEM (**A**) and SEM (**B**) images of Cu_2_O; SEM image of Cu_2_O-TCPP (**C**); TEM (**D**) and SEM (**E**) images of Cu–Cu_2_O; and SEM image of Cu–Cu_2_O-TCPP (**F**).

**Figure 2:**
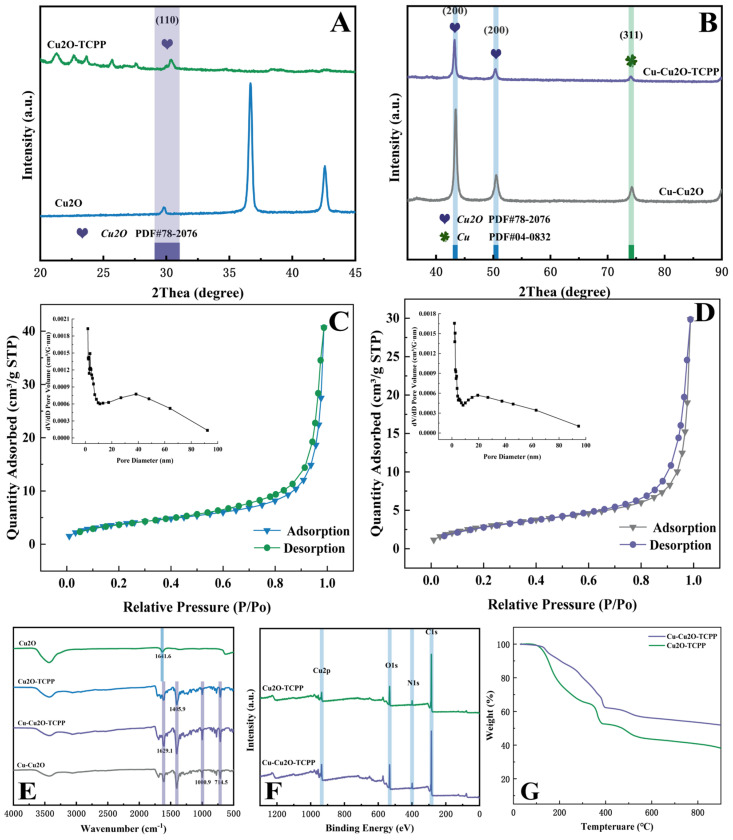
(**A**) XRD image of Cu_2_O-TCPP; (**B**) XRD image of Cu-Cu_2_O-TCPP; BET images of Cu_2_O-TCPP (**C**) and Cu-Cu_2_O-TCPP (**D**); (**E**) FTIR image of Cu_2_O, Cu-Cu_2_O, Cu_2_O-TCPP, and Cu-Cu_2_O-TCPP; (**F**) XPS total spectrum of Cu_2_O-TCPP and Cu-Cu_2_O-TCPP; (**G**) TGA image of Cu_2_O-TCPP and Cu-Cu_2_O-TCPP.

Firstly, the morphology and energy spectra of Cu_2_O, Cu-Cu_2_O, Cu_2_O-TCPP and Cu-Cu_2_O-TCPP were characterized by SEM. As shown in Figure 1, Cu_2_O has a globular structure, and Cu_2_O-TCPP has a two-dimensional laminar structure with protrusions on the surface, which indicates that the globular Cu_2_O is deposited onto the surface of TCPP. Cu-Cu_2_O is a chain-like structure, and Cu-Cu_2_O-TCPP has a two-dimensional laminar structure with irregular lamellar stacking with each other, which forms many voids and the area with the reactants. The peaks and response peak sizes of Cu and O elements can be observed from the Cu_2_O and Cu-Cu_2_O spectral spot maps, and the peaks and response peak sizes of C and N elements are also observed on the corresponding Cu_2_O-TCPP and Cu-Cu_2_O-TCPP spectral spot maps. Successful preparation of Cu_2_O-TCPP and Cu-Cu_2_O-TCPP was thus demonstrated. The FTIR spectra of Cu_2_O, Cu-Cu_2_O, Cu_2_O-TCPP and Cu-Cu_2_O-TCPP are shown in Figure 2E. The results showed that Cu_2_O at 1641.6 cm^−1^ and Cu-Cu_2_O, Cu_2_O-TCPP and Cu-Cu_2_O-TCPP at 1629.1 cm^−1^, 1405.9 cm^−1^, 1000.9 cm^−1^, and 714.5 cm^−1^, which correspond to the benzene ring backbone, C=O bond, and H-O bond, respectively, and the peak intensity of Cu-Cu_2_O-TCPP is higher than that of Cu-Cu_2_O. It can be proved that the interaction of Cu_2_O and TCPP, as well as that of Cu-Cu_2_O and TCPP, had interactions and formed coordination bonds, and Cu_2_O-TCPP and Cu-Cu_2_O-TCPP were successfully synthesized.

The XRD patterns of Cu_2_O, Cu-Cu_2_O, Cu_2_O-TCPP, and Cu-Cu_2_O-TCPP are shown in Figure 2A,B. The diffraction peaks of Cu_2_O and Cu_2_O-TCPP appeared at 2θ = 30°, which corresponded to the (110) crystal plane of the Cu_2_O crystals. The diffraction peaks of Cu-Cu_2_O and Cu-Cu_2_O-TCPP at 2θ = 43.3°, 50.5°, and 73.3°, which correspond to the (200) crystallographic plane of Cu_2_O and the (311) crystallographic plane of Cu, respectively, prove that Cu_2_O and Cu-Cu_2_O have been loaded onto the TCPP, respectively, and Cu_2_O-TCPP and Cu-Cu_2_O-TCPP have been successfully synthesized. Figure 2F shows the XPS test survey plots of Cu_2_O-TCPP and Cu-Cu_2_O-TCPP, which show the characteristic peaks of the valence states of Cu, O, N, and C elements. From the C1s spectrum (Figure S1), the characteristic peak at 284.1 eV corresponds to the C=C bond, and the characteristic peak at 288.2 eV corresponds to the O-C=O bond; the characteristic peak at 531.5 eV in the O1s spectrum (Figure S2) is due to the C-O bond. The characteristic peak at 934.8 eV in the spectrum of the element Cu (Figure S3) corresponds to Cu^2+^.

The BET of Cu_2_O-TCPP and Cu-Cu_2_O-TCPP are shown in Figure 2C,D. The rates of adsorption and desorption of Cu_2_O-TCPP and Cu-Cu_2_O-TCPP are slow at 0 < P/P0 < 0.8, and the rates of adsorption and desorption are drastic when the relative pressures are in the range of 0.8 < P/P0 < 1.0. From the data, it can be seen that the specific surface area of Cu_2_O-TCPP is 13.9671 m^2^·g^−1^, the pore volume is 0.062455 cm^3^·g^−1^, and the pore diameters of both adsorption and desorption are 18.0143 nm; and the specific surface area of Cu-Cu_2_O-TCPP is 10.9755 m^2^·g^−1^, the pore volume is 0.045949 cm^3^·g^−1^, and the pore diameters of both adsorption and desorption are 16.8304 nm. The large specific surface area of Cu_2_O-TCPP and Cu-Cu_2_O-TCPP was favorable for the adsorption of substances. Figure 2G shows the TGA characterization of Cu_2_O-TCPP and Cu-Cu_2_O-TCPP. The results showed that there was a slight weight loss of Cu_2_O-TCPP and Cu-Cu_2_O-TCPP at 30 °C–125 °C, which was mainly due to the evaporation of water and ethanol. The weight loss of 104 °C–418 °C was probably due to the breakage of the C=C bond and the C=O bond, and the weight loss rate gradually decreased after the temperature was higher than 450 °C. Finally, the residual fraction of Cu_2_O-TCPP was 38.3%; the residual fraction of Cu-Cu_2_O-TCPP was 51.92%.

### 3.2. Optimization of Reaction Conditions

In order to determine the optimal reaction conditions for the detection of AA by Cu_2_O-TCPP and Cu-Cu_2_O-TCPP, we optimized the concentration of Cu_2_O-TCPP and Cu-Cu_2_O-TCPP, the pH of the buffer solution and the concentration of PTA in the reaction system.

Figures S4–S6 show the optimized reaction conditions for Cu_2_O-TCPP. As shown in Figure S4, the R^2^ value of the standard curve between AA concentration 0–0.5 mM was the largest for Cu_2_O-TCPP concentrations of 0.45 mg/mL and 0.6 mg/mL, and the slope of the standard curve of 0.45 mg/mL was greater than that of 0.6 mg/mL. The larger the slope, the more obvious the change in fluorescence intensity is when the AA concentration changes the same, and the detection results are more accurate. Therefore, the concentration of Cu_2_O-TCPP of 0.45 mg/mL was chosen as the reaction concentration. As can be seen from Figure S5, the R^2^ values of the standard curves plotted at buffer pH = 6 and pH = 7.5 were maximum, and the slope of the standard curve was greater at buffer pH = 7.5, so the buffer of pH = 7.5 was selected as the pH for the reaction of this system. Figure S6 shows the results of PTA concentration optimization. From the results, the R^2^ values are the highest at PTA concentrations of 30 mM and 45 mM, and the slope of the standard curves was significantly greater at PTA 45 mM than that of PTA 30 mM. Finally, it was selected that 0.45 mg/mL of Cu-Cu_2_O-TCPP, PBS at pH = 7.5 and 45 mM PTA were selected as the optimum reaction conditions for Cu_2_O-TCPP.

The results of the optimization of the reaction conditions of Cu-Cu_2_O-TCPP are shown in Figures S7–S9. From the optimization results of Cu-Cu_2_O-TCPP concentration (Figure S7), the R^2^ value of the standard curve of AA was detected at the concentration of 0.45 mg/mL; the best linear fit of the curve was obtained in PBS solution at pH = 6.5 (Figure S8). The R^2^ value of the standard curve was highest when PTA = 20 mM, 30 mM, and 45 mM, and the slope of the standard curve is greatest at 45 mM (Figure S9). Therefore, 0.45 mg/mL of Cu-Cu_2_O-TCPP, PBS at pH = 6.5 and 45 mM PTA were selected as the optimum reaction conditions for Cu-Cu_2_O-TCPP.

### 3.3. AAO Activity

**Figure 3:**
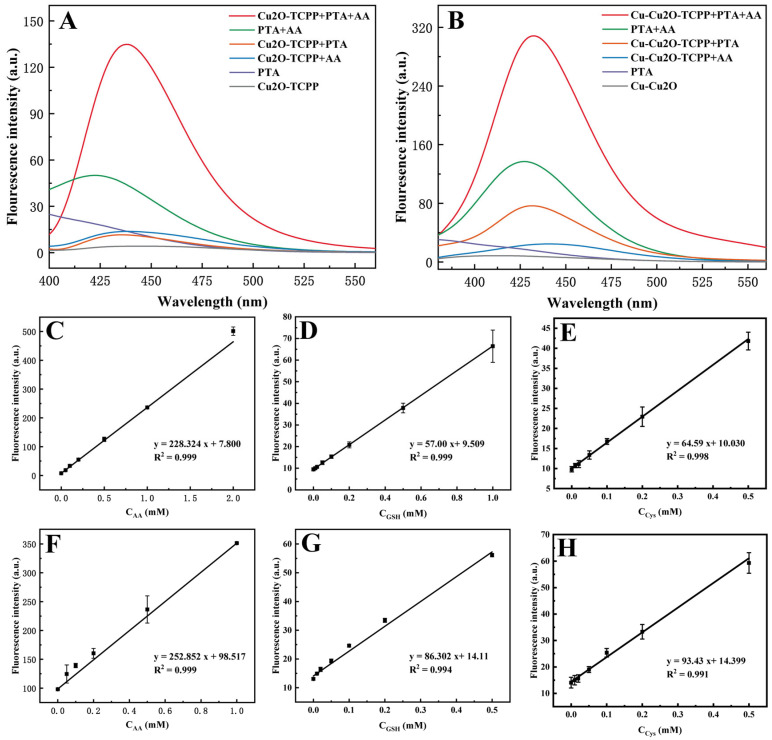
AAO nanozyme activity of Cu_2_O-TCPP (**A**) and Cu-Cu_2_O-TCPP (**B**); Cu_2_O-TCPP: linear relationship between AA (**C**), GSH (**D**), and Cys (**E**) concentrations and fluorescence intensity; Cu-Cu_2_O-TCPP: linear relationship between AA (**F**), GSH (**G**), and Cys (**H**) concentrations and fluorescence intensity.

PTA was used as a substrate to explore the AAO activity of Cu_2_O-TCPP and Cu-Cu_2_O-TCPP. After 15 min at 45 °C, no fluorescence was produced in the PBS buffer, PTA and AA reaction systems, while the reaction solution produced fluorescence after the addition of Cu_2_O-TCPP (Figure 3A) and Cu-Cu_2_O-TCPP (Figure 3B).

Under the optimal test conditions, the fluorescence intensity of the measured system with AA concentration is shown in Figure 3C,F. According to the detection results, the linear equation between AA concentration and fluorescence intensity was y = 228.324x + 7.800 and R^2^ = 0.999 for Cu_2_O-TCPP in the range of AA concentration 0–2 mM at Ex = 315 nm, and the linear equation between AA concentration and fluorescence intensity was y = 252.852x + 98.517 and R^2^ = 0.999 for Cu-Cu_2_O-TCPP in the range of AA concentration 0–1 mM. Subsequently, the linear relationships of glutathione and L-cysteine were established. Relevant linear equations, correlation coefficients and concentration ranges are displayed in Figure 3D–H.

### 3.4. Kinetic Analysis and Catalytic Mechanism

**Figure 4:**
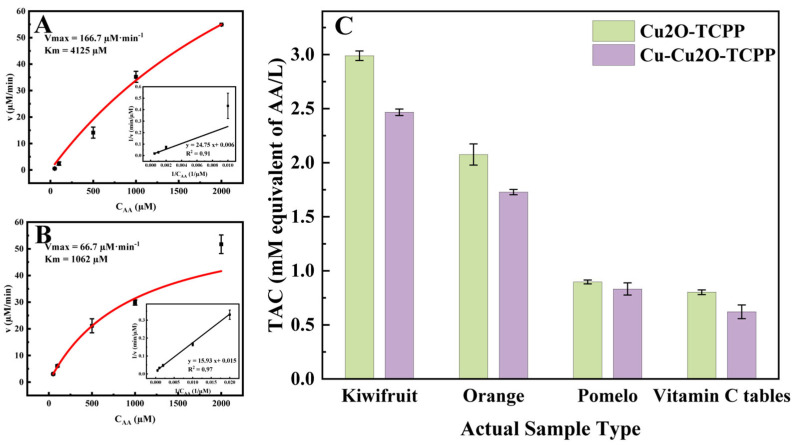
Kinetic analysis of Cu_2_O-TCPP (**A**) and Cu-Cu_2_O-TCPP (**B**); (**C**) actual sample testing.

Steady-state kinetic analysis of Cu_2_O-TCPP and Cu-Cu_2_O-TCPP was performed under optimal reaction conditions, and the enzymatic kinetic constants of Cu_2_O-TCPP and Cu-Cu_2_O-TCPP nanozymes, i.e., maximum reaction velocity (*Vmax*) and affinity for AA (*Km*), were obtained (Figure 4A,B). According to the results, the *Vmax* of Cu_2_O-TCPP was 166.7 mΜ/min, and the Km was 4125 μM. The Vmax of Cu-Cu_2_O-TCPP was 66.7 μM/min, and the *Km* was 1062 μM. A lower *Km* value indicates better interaction between the nanoenzyme and the substrate. The *Vmax* of Cu_2_O-TCPP is greater than that of Cu-Cu_2_O-TCPP, but Cu-Cu_2_O-TCPP has a greater affinity for AA.

In addition, the LODs for Cu-MOFs were 5.3 μM (Cu_2_O-TCPP) and 92.5 μM (Cu-Cu_2_O-TCPP), respectively. We compared (Table S2) with the previously reported nanoenzymes for TAC detection, indicating that Cu-MOFs have a wide detection range. Compared with Cu-Cu_2_O-TCP, Cu_2_O-TCPP has a wider detection range and a lower detection limit.

### 3.5. Actual Samples

In order to verify the accuracy of Cu_2_O-TCPP/Cu-Cu_2_O-TCPP for TAC detection, the content of AA in vitamin C tablets purchased in pharmacies was detected by the method and titration method of this study. The results of titration showed that the TAC content of 0.176 g/L vitamin C tablet solution was 0.845 AA//L, and the detection results of this method are shown in Figure 4C. The TAC content of 0.176 g/L vitamin C tablet solution was 0.815 AA/L for Cu_2_O-TCPP, and the TAC content for Cu-Cu_2_O-TCPP was 0.621 AA/L. Compared with the traditional method, the error rate of Cu_2_O-TCPP is only 3.6%, and the detection result is accurate.

In addition, the TAC content of three fruits, kiwifruit, orange and grapefruit, was also tested. The TAC content results of kiwifruit, orange and grapefruit are shown in Figure 4C. The detection results of Cu_2_O-TCPP were 3.0 mM, 2.1 mM and 0.9 mM equivalent AA//L, respectively, and the detection results of Cu-Cu_2_O-TCPP were 2.5 mM, 1.7 mM and 0.8 mM equivalent AA/L, respectively. The antioxidant capacity of kiwifruit is higher than that of oranges, and the antioxidant capacity of oranges is higher than that of grapefruit, as in previous studies. Therefore, Cu_2_O-TCPP and Cu-Cu_2_O-TCPP nanozymes were used for the detection of TAC in fruits, and the results were accurate and reliable.

## 4. Conclusions

In this study, two two-dimensional Cu-MOFs nanozymes with AAO and POD enzyme activities were synthesized to obtain a fluorescent detection method for total antioxidant capacity in fruits. Cu-MOFs can catalyze AA oxidation and produce ·OH, meanwhile ·OH oxidizes PTA under the POD activity of Cu-MOFs to produce fluorescence, and the higher the AA concentration, the stronger the fluorescence intensity within a certain concentration range. Therefore, an efficient and portable fluorescence sensing detection platform for the detection of total antioxidant capacity in food was successfully established. This fluorescence sensing platform effectively avoids the shortcomings of easy inactivation of natural enzymes. The experimental results showed that the platform had good linear detection performance of reducing substances such as ascorbic acid, glutathione and L-cysteine, and showed high accuracy in the detection of vitamin C tablets and fruit samples, especially Cu_2_O-TCPP nanozyme. This provides a new idea and technical means for the rapid and accurate assessment of total antioxidant capacity in food, and is expected to be widely used in the field of food safety and nutritional value assessment.

## Data Availability

The original contributions presented in this study are included in the article/Supplementary Material. Further inquiries can be directed to the corresponding authors.

## Supplementary Materials

The following supporting information can be downloaded at: https://www.mdpi.com/article/10.3390/nano16110665/s1. Refs. [65,66,67,68,69,70] are cited in the Supplementary Material file.
