# Supplementary material for: Cu-MOFs Nanozymes with Ascorbate Oxidase and Peroxidase-like Activity for Sensitive Fluorometric Detection of Total Antioxidant Capacity in Fruits
Source: Nanomaterials (Basel). 2026 May 25;16(11):665. doi: 10.3390/nano16110665 (PMC13258385; doi:10.3390/nano16110665)
Supplement: Supplementary file 1 [file nanomaterials-16-00665-s001.zip › nanomaterials-4281246-supplementary.pdf]

Electronic Supplementary Information

Cu-MOFs Nanozymes with Ascorbate Oxidase and Peroxidase-Like Activity for Sensitive Fluorometric Detection of Total Antioxidant Capacity in Fruits

Yanyan Huang[b 1], Jing Chen[b 1], Ai Nasi[c 1], Yiming Zhao[b], Xin Ding[b], Dan Xu[b], Fengzhi Lyu[b], Donghui Xu[b a*], Meng Zhang[b], Ge Chen[b], Guangyang Liu[b a*]

[a] National Center of Technology Innovation for Comprehensive Utilization of Saline-Alkali Land, 8 Zhihui Road, Agricultural High tech Industry Demonstration Zone, Yellow River Delta, Dongying, Shandong Province, China 257347

[b] State Key Laboratory of Vegetable Biobreeding, Institute of Vegetables and Flowers, Chinese Academy of Agricultural Sciences, Key Laboratory of Vegetables Quality and Safety Control, Ministry of Agriculture and Rural Affairs of China, Beijing 100081, China

[c] Key Laboratory of Geriatric Nutrition and Health, Ministry of Education, Beijing Technology & Business University, Beijing 100048, China

[*]Corresponding authors:

E-mail addresses: xudonghui@caas.cn, liuguangyang@caas.cn

Figure S1

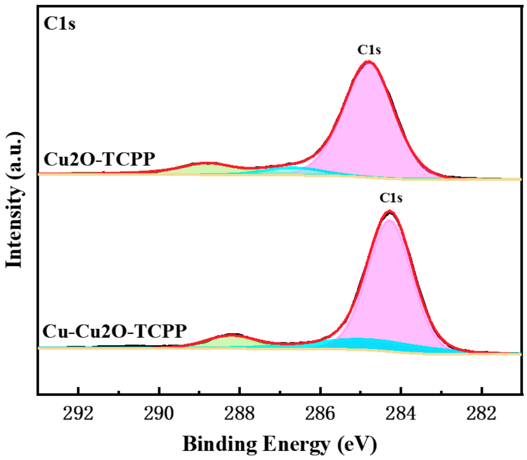

Figure S1. XPS C1s spectrum of Cu_2O-TCPP and Cu-Cu_2O-TCPP.

Figure S2

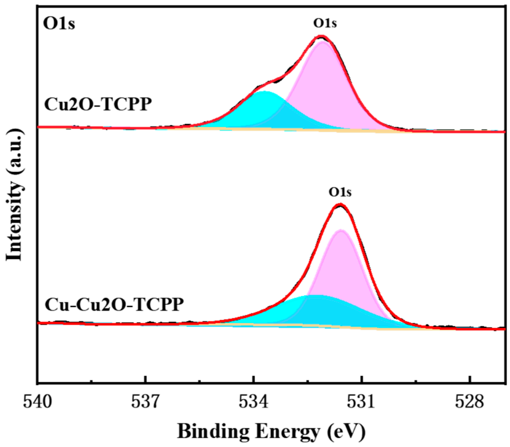

Figure S2. XPS O1s spectrum of Cu_2O-TCPP and Cu-Cu_2O-TCPP.

Figure S3

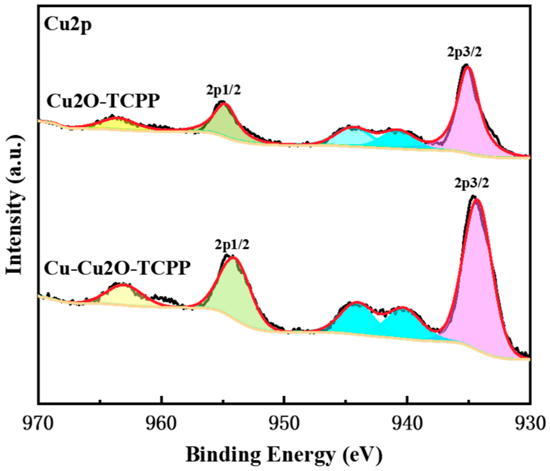

Figure S3. XPS Cu2p spectrum of Cu_2O-TCPP and Cu-Cu_2O-TCPP

Figure S4

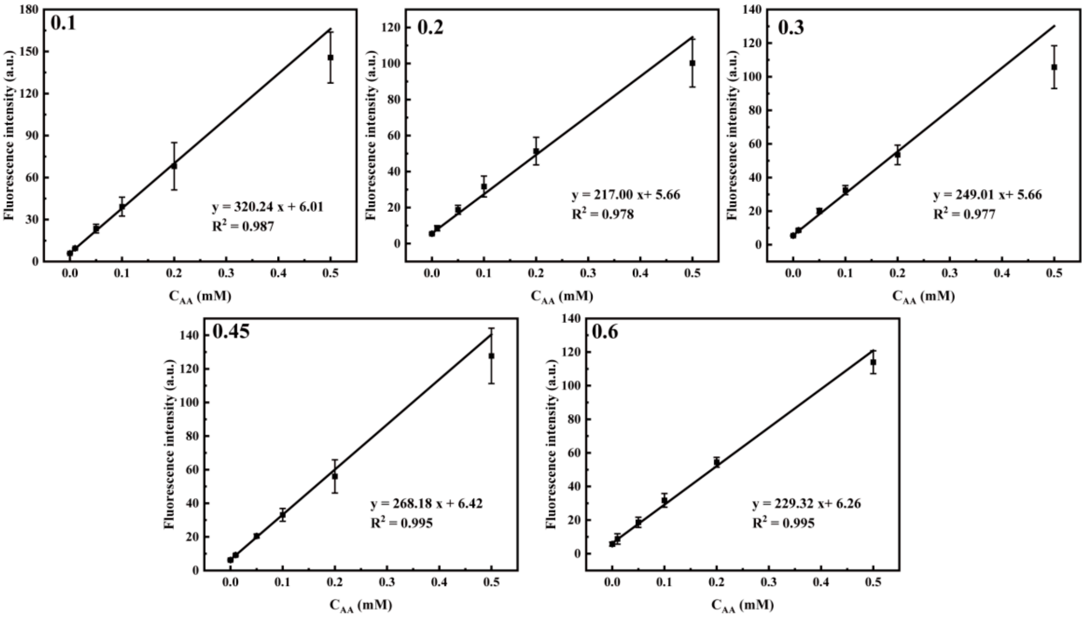

Figure S4. Optimization of Cu_2O-TCPP concentration.

Figure S5

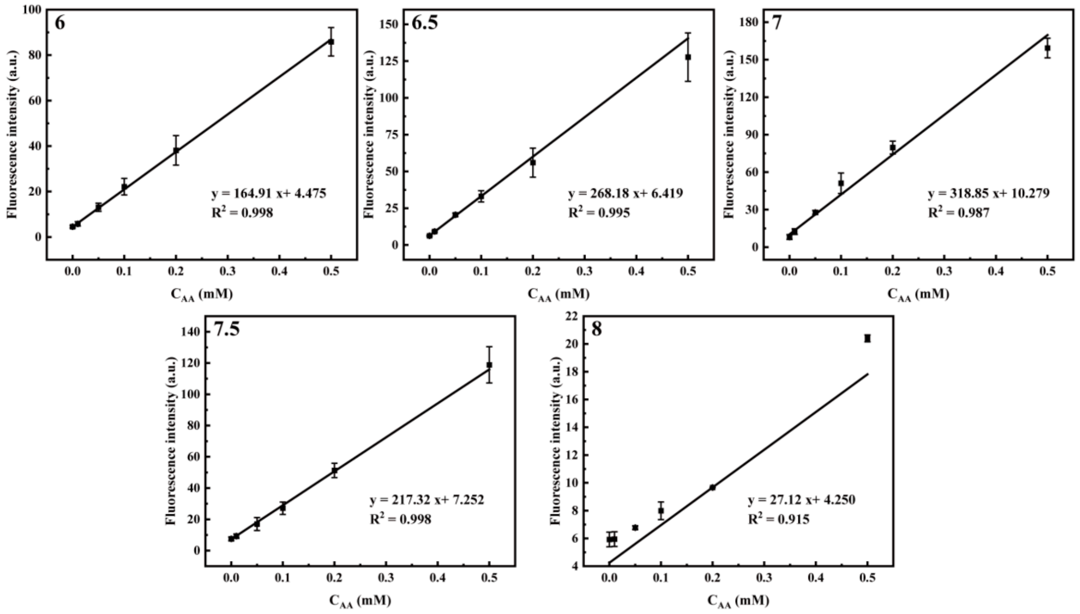

Figure S5. Reaction pH optimization of Cu_2O-TCPP**.**

Figure S6

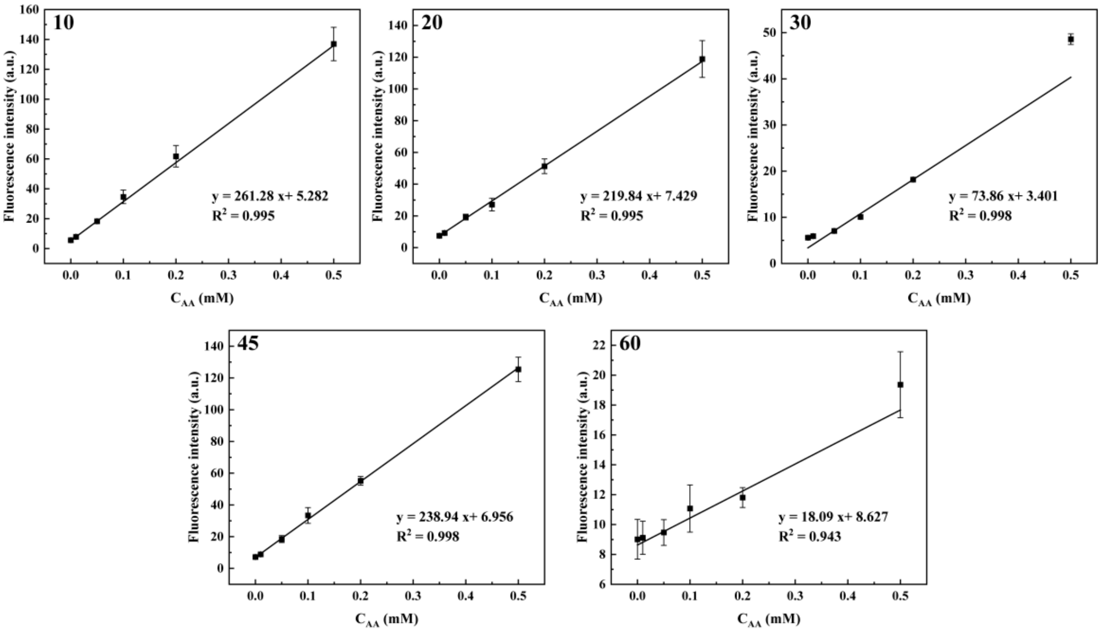

Figure S6. PTA concentration optimization of Cu_2O-TCPP.

Figure S7

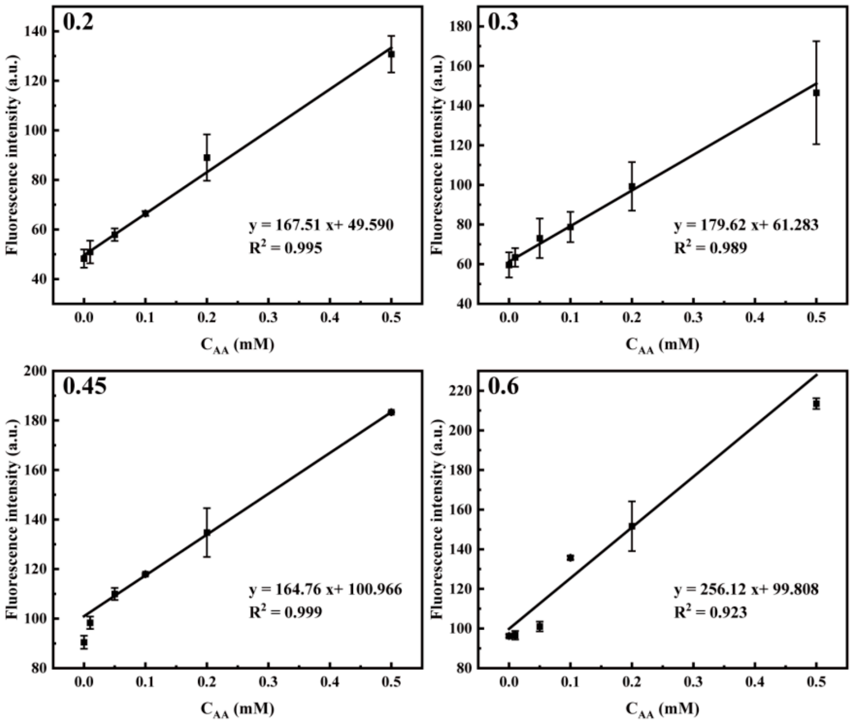

Figure S7. Optimization of Cu-Cu_2O-TCPP concentration.

Figure S8

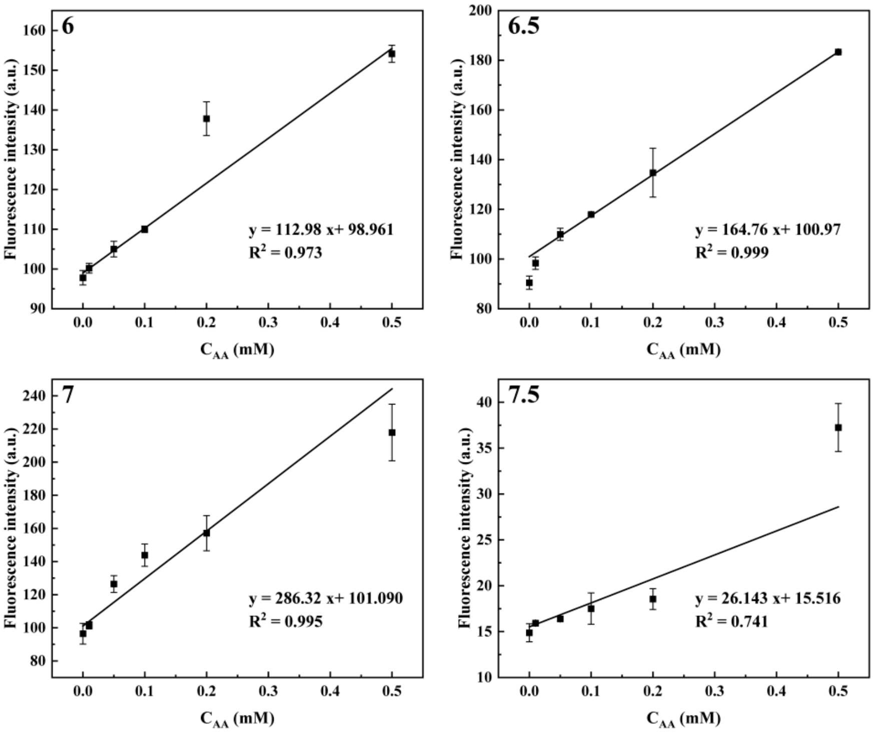

Figure S8. Reaction pH optimization of Cu-Cu_2O-TCPP.

Figure S9

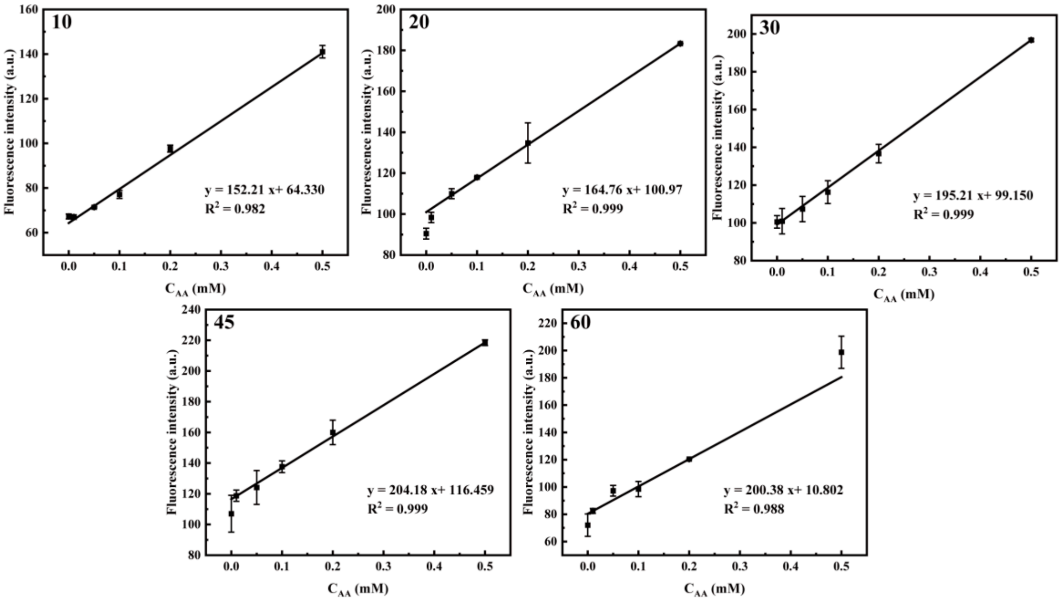

Figure S9. PTA concentration optimization of Cu-Cu_2O-TCPP.

Table S1

Table S1 Reagents and instruments

Name	Instrument model / Reagent purity	Manufacturer
Ultrapure water systems	Milli-Q Advantage	Milibauth USA
Electronic balances	PL203, AL204	METTLER TOLEDO
Manual pipettes	20 μL-5 mL	Eppendorf AG
pH meter	PHS-3C	Shanghai INESA Scientific Instrument Co., Ltd.
Freeze dryer	LGJ-10N	Beijing Yaxing Instrument Technology Co., Ltd.
Tabletop high-speed refrigerated centrifuge	3K15	Beijing Tianlin Hengtai Technology Co., Ltd.
Collector type thermostatic heating magnetic stirrer	DF-101S	Shaanxi Taikang Biological Technology Co., Ltd.
Fluorescence spectrophotometer	RF-5301PC	Shimadzu Enterprise Management (China) Co., Ltd.
p-Phthalic acid	≥99%	Sinopharm Chemical Reagent Co., Ltd.
$CuSO_4 \cdot 5H_2O$ (Cupric sulfate)	≥99%	Beijing Chemical Works
$C_6H_5Na_3O_7$ (Sodium citrate)	≥98%	Suzhou Ruri Chemical Technology Co., Ltd.
NaOH (Sodium hydroxide)	≥96%	Tianjin Huihang Chemical Technology Co., Ltd.
$C_6H_8O_6$ (L-Ascorbic acid)	≥99.7%	Xilong Chemical Co., Ltd.
C_2H_6O (Ethanol)	≥99%	Sinopharm Chemical Reagent Co., Ltd.
PBS (Phosphate buffered saline)	-	Solarbio
$CuCl_2$ (Cupric chloride)	≥99%	Sinopharm Chemical Reagent Co., Ltd.
$NaBH_4$ (Sodium borohydride)	≥99%	Jiangsu Runfeng Synthesis Technology Co., Ltd.
C_3H_7NO (N, N-Dimethylforma mide)	≥99%	Shanghai Macklin Biochemical Technology Co., Ltd.
$C_{48}H_{30}N_4O_8$ (4,4,4,4-（Porphine-5,10,15,20-tetrayl))	≥97%	Shanghai Macklin Biochemical Technology Co., Ltd.
$C_7H_6O_2$ (Benzoic acid)	≥99.5%	Sinopharm Chemical Reagent Co., Ltd.
Kiwifruit	-	Meituan Little Elephant Supermarket
Orange	-	Meituan Little Elephant Supermarket
Pomelo	-	Meituan Little Elephant Supermarket
Vitamin C tables		Drug store

Table S2

Table S2 Comparison of methods

Nanozymes		Method	Enzyme activity	LOD/AA	Detection range	Km/TMB	References
Cu-MOFs	Cu_2O-TCPP	Fluorescence	AO and POD	5.3 µM	0-2 mM	4.125 mM	This work
	Cu-Cu_2O-TCPP	Fluorescence		92.5 µM	0-1 mM	1.062 mM	
MOF	Ni–Fe MOFs	Colorimetry	POD	0.094 µM	0-90 µM	2.22 mM	(Liu, Song, Zhang, & Shang, 2024)
	HS-Mn/Co-MOF	Colorimetry	Oxidase	3 nm	0.01-120 µM	0.15 mM	(Liang et al., 2025)
	Mn-NiO	Colorimetry	Oxidase	0.028 µM	0.04-60 µM	0.074 mM	(He, Feng, Zhang, & Huang, 2025)
Others	BSA-Au NCs	Fluorescence	Oxidase	0.4 µM	3-50 µM	—	(Ni et al., 2021)
	$MnSiO_3$	Fluorescence	Oxidase	0.057 µM	0.1-50 µM	—	(Huang, Lv, Zhang, Wang, Zhang, & Fan, 2025)
	Fe-CDs	Fluorescence	POD	2.1 µM	4-40 µM	—	(Li et al., 2025)
		Colorimetry		0.4 µM	6.3-150 µM	—	

References

He, Y., Feng, M., Zhang, X., & Huang, Y. (2025). MOF-derived bundle-like Mn doped NiO

with rich oxygen vacancy as oxidase mimic for the determination of total antioxidant capacity. Sensors and Actuators B: Chemical, 428, 137227. https://doi.org/https://doi.org/10.1016/j.snb.2025.137227.

Huang, S., Lv, X., Zhang, Y., Wang, J., Zhang, X., & Fan, D. (2025). When MnSiO3 meets ratiometric fluorescence: A facile, cost-effective ratiometric fluorescent platform based on oxidase-like MnSiO3 nanozyme for versatile Total Antioxidant Capacity (TAC) measurements. Sensors and Actuators B: Chemical, 423, 136858. https://doi.org/https://doi.org/10.1016/j.snb.2024.136858.

Li, J., Li, Y., Shi, Y., Li, Z., Deng, C., Zhang, G., . . . Yu, X. (2025). Fluorescence/colorimetric intelligent sensor based on Fe-adsorbing carbon dots nanozymes for selective recognition and quantification of total antioxidant capacity in foods and cells. Food Chemistry, 490, 145052. https://doi.org/https://doi.org/10.1016/j.foodchem.2025.145052.

Liang, T., Huang, Y., Yang, L., Chai, Y., Hao, Z., Chen, H., & Ma, G. (2025). Hollow Mn/Co-MOF as a Powerful Oxidase-Like Nanozyme for Detection of Total Antioxidant Capacity and Black Tea Fermentation Degree. 21(12), 2411275. https://doi.org/https://doi.org/10.1002/smll.202411275.

Liu, A., Song, W., Zhang, C., & Shang, H. (2024). Colorimetry/Smartphone Dual-Mode Sensing Platform Based on Nanorod-Shaped Ni–Fe MOFs for Ascorbic Acid Detection. ACS Applied Nano Materials, 7(11), 13400-13406. https://doi.org/10.1021/acsanm.4c01873.

Ni, P., Liu, S., Wang, B., Chen, C., Jiang, Y., Zhang, C., . . . Lu, Y. (2021). Light-responsive Au nanoclusters with oxidase-like activity for fluorescent detection of total antioxidant capacity. Journal of Hazardous Materials, 411, 125106. https://doi.org/https://doi.org/10.1016/j.jhazmat.2021.125106.
